# Radiocopper in Radiopharmacy and Medical Use: Current Status and Perspective

**DOI:** 10.1021/acs.jmedchem.4c02885

**Published:** 2025-02-03

**Authors:** Paweł Kręcisz, Katarzyna Stefańska, Jakub Studziński, Monika Pitucha, Agnieszka Czylkowska, Paweł Szymański

**Affiliations:** † Department of Pharmaceutical Chemistry, Drug Analyses and Radiopharmacy, Faculty of Pharmacy, Medical University of Lodz, Muszyńskiego 1, 90-151 Lodz, Poland; ‡ Independent Radiopharmacy Unit, Faculty of Pharmacy, Medical University of Lublin, Chodźki 4a, 20-093 Lublin, Poland; § Institute of General and Ecological Chemistry, Faculty of Chemistry, Lodz University of Technology, Żeromskiego 116, 90-924 Lodz, Poland; ∥ Department of Radiobiology and Radiation Protection, Military Institute of Hygiene and Epidemiology, Kozielska 4, 01-163 Warsaw, Poland

## Abstract

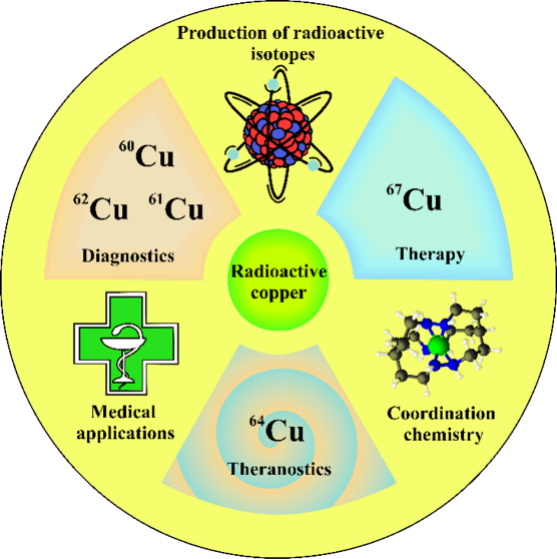

Of the 32 known copper isotopes, some have interesting properties for nuclear medicine, for example the short-lived ^60^Cu, ^61^Cu, ^62^Cu, the moderate long-lived ^64^Cu and the long-lived ^67^Cu. Due to their emission properties, copper isotopes are suitable for both imaging diagnostics (^60^Cu, ^61^Cu, ^62^Cu, ^64^Cu) and targeted radiotherapy (^64^Cu and ^67^Cu). As their chemical properties are virtually identical, a single radiopharmaceutical structure can be labeled with different isotopes, depending on the clinical application. This, combined with the ability to combine radioisotopes with different nuclear properties with the same ligand, makes them extremely versatile. The purpose of this review is to introduce the world of copper radiopharmaceuticals and to summarize recent advances in methods for producing copper radioisotopes and the preclinical research of radiopharmaceuticals labeled with copper radioisotopes.

## Significance

- The theranostic potential of copper isotopes opens up the possibility of using a wide range of diagnostic carriers for therapeutic purposes by modifying only the isotope with which the molecule is labeled.
- Work on radiocopper-labeled ligands is one of the most important areas of research in nuclear medicine that has a unique impact on the development of this field.
- The challenges in developing new radiopharmaceuticals labeled with radiocopper are strongly related to the production of copper isotopes.

## Introduction

Nuclear medicine is currently one of the fastest-growing areas responding to the challenges of modern Medicine.^1,2^ Thanks to constantly developing techniques, radioactive isotopes form a valuable part of diagnostic and therapeutic tools in many areas of medicine.^3^ Cancer diagnostics and therapy have an extensive range of isotope tracers at their disposal,^4^ which are used to recognize and treat *inter alia* lymphomas, lung cancer, breast cancer, prostate cancer and neuroendocrine tumors (NETs).^5−7^ In neurology, radiotracers are selected according to a given disease such as brain tumors, epilepsy, Parkinson’s disease or Alzheimer’s disease.^8−11^ Radioactive isotopes are used to diagnose diseases of the bones, kidneys, nervous system, heart and vessels, as well as the thyroid and liver.^12−14^ Radiopharmaceuticals have applications in cancer therapy, especially thyroid cancer and prostate cancer.^15,16^

The use of a radiopharmaceutical for diagnostic or therapeutic purposes is determined based on the type of radiation emitted and its half-life. Radioisotopes with a short physical half-life are used in clinical practice; these usually range from a few minutes to several hours or several days. In the development of new radiopharmaceuticals, radionuclides with higher atomic numbers and longer half-lives have also been studied for both imaging and therapeutic purposes, including halogens (^124^I, ^120^I, ^76^Br) and a number of metals (^177^Lu, ^90^Y, ^68^Ga, ^111^In). Nonstandard radiometals are being tested (e.g., ^64^Cu, ^89^Zr), which have great potential but also numerous limitations.^17^

Generally, emitters of α, β^–^ particles and Auger electrons, i.e., particles depositing high energy over a short distance, are used for therapeutic purposes. A common use is in the treatment of thyroid cancer, where a synthetic isotope of iodine, ^131^I, has long been used, and in the treatment of cancer metastases to bones using phosphorus ^32^P, strontium ^89^Sr, rhenium ^186^Re, ^188^Re and samarium ^153^Sm.^18,19^ In contrast, γ or β^+^ radiation emitters such as technetium (^99m^Tc), iodine (^131^I, ^123^I), fluorine (^18^F), and gallium (^68^Ga) are used for diagnostic purposes. Interesting results can be obtained when labeling cholinesterase inhibitors with ^68^Ga; these are used for positron emission tomography (PET) imaging of changes in the level of acetyl- and butyrylcholinesterase in neuronal synaptic spaces, which plays an important part in the diagnosis of neurodegenerative diseases such as Alzheimer’s disease (AD).^20^

For most targeted radiopharmaceuticals, the radiotracer is crucial. It is bound to the pharmacophore by a bifunctional chelating agent (BFCA), which forms a stable covalent bond between the tag and the targeting ligand and ensures stable metal complexation. The type of radionuclide determines the complexation ability, level of radiotoxicity and potential use in nuclear medicine. Additionally, the use of radiometal makes it possible to replace a diagnostic agent with a therapeutic one, fitting into the theranostics scheme.

Copper is particularly interesting in this regard as, being a trace element, it takes part in several key enzymatic reactions in the human body. It plays an important role in angiogenesis and can stimulate endothelial cell proliferation in a variety of benign and malignant situations. The cellular distribution of copper is quite complex and although precise mechanisms are known, recent data on how copper is internalized by human cells remain unclear; however, it is known that copper in its ionic form (Cu^2+^) binds rapidly to plasma proteins in the blood (albumin, ceruloplasmin, transcuprein), thus allowing it to reach the cell surface.^21^

Copper ions are capable of forming complexes with many types of ligands. Their selection for treatment is dependent on several factors, but the most important is the direction of biological activity of the formed complex compounds. A number of studies have examined the coordination connections of ligands with the copper atom, and these have yielded in valuable findings.^22^

Copper isotopes are stabilized to a great degree by ligands, which facilitates their use in imaging and treatment. By forming stable and neutral complexes with the radioactive copper ion, the ligand can safely deliver them to targeted tissues. The specific coordination chemistry of chelators enables precise manipulation of the properties of the copper complex, thus optimizing its biological activity.^23^ The chemistry underlying copper radiopharmaceuticals continues to evolve, expanding their use in targeted therapies and imaging, indicating the potential for new developments in both clinical and research environments.^24^ While the use of copper isotopes in pharmaceuticals shows great promise, challenges remain in optimizing ligand design and understanding the full scope of their biological interactions.

Due to their emission properties, copper isotopes are suitable for both imaging diagnostics (^60^Cu, ^61^Cu, ^62^Cu, ^64^Cu) and targeted radiotherapy (^64^Cu and ^67^Cu). The most promising radioisotope seems to be ^64^Cu due to its longer half-life (12.7 h) and commercial availability. The applications of copper radioisotopes can be expanded with the possibility of beta-emitting radioligand therapy with ^67^Cu through the formation of so-called *theranostic pair*. The premise of theranostics is to use of diagnostic tools to tailor specific treatments for individual patients. The use of a specific diagnostic test identifies a specific molecular target on cancer cells, allowing the therapeutic agent to bind specifically to receptor sites. The similar chemical properties shared by copper isotopes allow for the attachment of different radionuclides to the same type of vector, thanks to which therapeutic and diagnostic radiopharmaceuticals have identical pharmacokinetics in the human body.^25^

## Production of Medically Interesting Copper Radionuclides

Copper radionuclides can be produced in a variety of different ways. New production routes based on charged particles, neutrons, or isotope generators have been established in recent years.^25,26^ For some copper radioisotopes with potential medical value, novel methods such as photonuclear activation have also been reported.^27−29^ The main routes for producing the copper isotopes used in nuclear medicine are summarized in Table 1.

**Table 1:** Typical Production Routes of Copper Radioisotopes

isotope	typical production routes
^60^Cu	cyclotron
^61^Cu	cyclotron

^62^Cu	generator
	cyclotron

^64^Cu	cyclotron
	nuclear reactor

^67^Cu	nuclear reactor
	cyclotron
	photonuclear reaction

The applicability of a given approach depends on the availability of the projectile at an appropriate energy range at the production site. Different methods produce copper radioisotopes with varying yields, some of which that may be too low for clinical-scale production. However, even those routes can prove valuable for research and development of targetry, chemical separation methods, novel tracers or preclinical studies. Another important factor is the choice of the target material. Naturally abundant materials are useful for initial method development, but usually are inapplicable in large-scale production. Nearly all methods benefit from using enriched target materials, but their cost may limit their potential use. Different routes also lead to products with varying chemical, radiochemical and radionuclidic purity. While chemical purity can be improved by optimizing the separation method, radiochemical impurities can lead to issues with personnel safety and waste treatment. As radioisotopic impurities cannot be separated, optimization of the production method is crucial.^30^

### Isotope ^64^Cu

^64^Cu has unique decay properties, as it has three modes of decay: β^+^ (19%; Eb^–^ 278 keV), electron capture (42.5%), and β^–^ (38%: Eb^–^ 190 keV) decay. Its decay is also accompanied by emission of Auger electrons.^31^ It has a moderate half-life of 12.7 h, compatible with the kinetics of carriers such as antibodies and peptides. Its positron emission has a low energy, allowing high resolution imaging without abundant gamma emissions impairing the image. Emitted positrons have short-range, resulting in spatial resolution comparable to ^18^F.^32^ Its β^–^ particles and Auger electrons have sufficient cytotoxic potency, making it suitable for targeted radionuclide therapy.^24^ The γ rays associated with ^64^Cu decay (0.511 MeV, 35.6%; 1,35 MeV, 0.6%) have not been investigated for single-photon emission computed tomography (SPECT) imaging as the suitable energy range falls between 100 and 200 keV.^30^ As such, ^64^Cu is a highly versatile isotope, with uses in both PET diagnostics and radiotherapy. Multiple decay schemes also allow quantitative PET imaging to calculate dosimetry prior to targeted radiotherapy, either with ^64^Cu or ^67^Cu.^33^

There are several routes for producing ^64^Cu with varying yields, specific activities, radionuclide, radiochemical and chemical purities. It is most commonly produced either in a nuclear reactor or a cyclotron. Low specific activity ^64^Cu can be produced in a nuclear reactor in ^63^Cu(n, γ) ^64^Cu, while the high specific activity form can be achieved via ^64^Zn(n, p) ^64^Cu reactions. The ^63^Cu(n, γ) ^64^Cu reaction can be performed with thermal neutrons, while ^64^Zn(n, p) ^64^Cu requires fast neutrons.^34^

^64^Cu in a noncarrier-added form can be produced with high specific activity by the ^64^Zn(n, p) ^64^Cu process using ^nat^Zn or an enriched ^64^Zn target. Due to the small cross-section of the reaction, achieved with fission neutrons (34.72 ± 6.82 mb), the yield is quite small.^35^ Irradiation of a target with natural composition also leads to the formation of ^63^Ni, ^69m^Zn, and ^67^Cu, which contaminate the final product. However, because of the longer half-life of ^67^Cu, its contamination level rises with time, restricting the window in which the ^64^Cu product is usable.^36^ Enrichment of the target can double the ^64^Cu production, while suppressing these contaminants. Regardless of the target used, water moderated reactors have a higher thermal neutron flux than fast neutron flux, resulting in the production of long-lived ^65^Zn (*T*_1/2=_ 245 d) via the ^64^Zn(n, γ) ^65^Zn reaction, induced by the thermal part of neutron spectrum. ^65^Zn builds up in the target, making target processing and repurposing much more difficult. However, this can be improved by shielding the target with a material with high neutron capture cross-section, such as boron, cadmium or hafnium; indeed, boron nitride shielding has been reported to reduce ^65^Zn production by 2 orders of magnitude.^37^ Nevertheless, ^64^Cu with a specific activity (12.2 ± 0.8 GBq/μg) comparable to that achieved by cyclotron-based routes can be achieved in quantities (670.9 ± 2.5 MBq) suitable for medical use, thus filling any gaps in supply.^38^

Direct activation of natural copper by thermal neutrons via the ^63^Cu(n, γ) ^64^Cu reaction produces large quantities of ^64^Cu; however, the product has low specific activity (∼3 GBq/μg) as it is not possible to chemically separate the radioactive copper from the carrier, making it unsuitable for labeling targeted molecules.^34^ However, the produced ^64^Cu can be used in the [^64^Cu]Cl_2_ form as a standalone radiopharmaceutical for PET diagnostics any concern regarding “cold” copper toxicity. The produced ^64^Cu decays to ^64^Ni and ^64^Zn, which can lead to the formation of, respectively, ^65^Ni (*T*_1/2_ = 2.5 h) and ^65^Zn. The ^65^Cu present in the target can be activated to ^66^Cu (*T*_1/2_ = 5.1 min). While all of these isotopes contaminate the product, only ^65^Zn can be a cause for concern, because ^65^Ni and ^66^Cu decay out.^39^ The yield of the process can also be further improved upon by irradiating copper nanoparticles instead of bulk material.^40^ Another modification that can increase the specific activity of the product is to use the Cu-phthalocyanine complex to take advantage of the Szilard–Chalmer effect; however, the specific activity of the product is still orders of magnitude lower than the one of the obtained in fast neutron capture because of the formation of unchelated Cu atoms by gamma radiolysis of phthalocyanine.^41^ This method can be used to produce ^64^Cu at a low cost in countries with limited cyclotron facilities; this is important as according to the International Atomic Energy Agency (IAEA) database, 79 research reactors are utilized for isotope production around the world in 2020^42^ and 81 are currently in operation.^43^

In recent years, the production of ^64^Cu using accelerator-driven neutron sources has been investigated via the ^64^Zn(n,p) ^64^Cu and ^65^Cu(n, 2n) ^64^Cu reactions.^44^ Accelerator-driven neutron sources operate by irradiating a light element, such as carbon and beryllium^45^ or deuterium and tritium with a deuteron beam.^44^ Such machines are rare,^46^ but produce neutrons with higher energies than nuclear reactors, exploiting the higher cross-section values of nuclear reactions achievable in higher energy ranges. The previously mentioned ^64^Zn(n,p) ^64^Cu reaction reaches a maximum cross section of 250 mb at 11 MeV, which is around four times higher than the cross-section reported in nuclear reactors. The ^65^Cu(n, 2n) ^64^Cu reaction has a threshold of 10 MeV and reaches an almost constant value of 850 mb at 14 MeV. It is estimated that by using a neutron source with a higher neutron emission rate, 10.2 GBq and 35 GBq of ^64^Cu can be produced in (n, 2n) and (n, p) reactions, respectively.^44^

Generally, production of ^64^Cu is focused on the ^64^Ni(p, n) ^64^Cu reaction, performed on isotopically enriched nickel targets in low-energy cyclotrons.^47^ While another approach based on the use of deuteron beams, exploiting the ^64^Ni(d, 2n) ^64^Cu channel has been proposed,^48^ it has not been widely adopted. The ^64^Ni(p, n) ^64^Cu reaction reaches its maximum cross-section at 11.5 MeV proton energy, making it expedient in low-energy medical cyclotrons.^49^ Above 5 MeV proton energy, ^61^Co (*T*_1/2_ = 1.65 h; 100% β^–^) is coproduced via the ^64^Ni(p, α) ^61^Co reaction, which necessitates a waiting period for the cobalt radioisotope to decay out before the target material can be safely recycled; alternatively the ^61^Co can be separated in quantities useful for small animal studies.^50^ The process typically involves electroplating a ^64^Ni target on a gold disc at a thickness of >100 μm up to 750 μm,^49,51,52^ however thinner targets have also been utilized (about 10 μm^33^). The target is then bombarded with protons with 11 to 14 MeV incident energy.^33,49,52,53^ After the irradiation, the target is dissolved and the ^64^Cu is separated from the ^64^Ni and contaminants using an anion exchange column. The ^64^Ni target can be then recycled.^52^ Alternatively, a cation-exchange resin eluted with HCl/acetone solution can be employed, yielding ^64^Cu with high chemical purity.^53^ In a 2022 IAEA publication, production yields of ^64^Cu via this reaction were reported to reach 100 MBq/μAh,^54^ but yields exceeding that value (131 ± 40 MBq/μAh) have also been demonstrated.^55^ Recently, production routes based on proton irradiation of solid ^64^Ni targets, utilizing a commercially available, automated system, have also been developed.^49,55^

An important development in this area is the application of liquid target systems,^56^ i.e., irradiating a solution containing dissolved salts of the target nuclei instead of using more conventional electroplated targets. The most notable disadvantages of solid targets include the need for time-consuming electroplating procedures, the quality of electroplating influencing the yield, as well excessive heat generated by the beam current causing possible target melting and evaporation,^52^ and the complex and lengthy procedures of target recycling, which further increase the manufacturing cost. Furthermore, when working with a solution of the target material the irradiated solution can be handled with automated systems to simplify the process. Finally, the solution can be purified and the labeled radiopharmaceutical synthesized in only 1 h after the end of bombardment, which is a significant improvement over the solid target technique. It is also possible to optimize production costs by individually calculating the amount of the costly enriched ^64^Ni and its irradiation time for each production. It should be noted, however, that utilization of liquid targets for production of radiometals comes with an unique set of challenges and warrants further research. Most notable issues include in-target precipitation and radiolysis of water, leading to gas evolution and a dangerous pressure rise inside of the target enclosure.^57^ Moreover, accelerated degradation of foils, used as beam windows, sometimes leading to their rupture, has also been reported.^58,59^ Additionally, a single preparation of the enriched target solution is enough to satisfy tens of productions. Although the yields achieved with this method are generally lower than those achieved with the conventional process (up to 14 MBq/μAh of ^64^Cu), the approach allows for production of sufficient activities for distribution to other PET centers.^59^ Another potential advantage is that both the preparation procedure and the equipment are very similar to those used in existing processes for the production of ^18^F from ^18^O enriched water, which could allow for the simple adoption of this technique in existing facilities.^54^ Additionally, production of ^64^Cu via the ^64^Ni(n, p) ^64^Cu reaction, using a high-power laser-driven accelerator has been proposed. With an existing 150 TW laser and appropriate targetry, activities suitable for preclinical studies could be achieved^60^

Due to the high cost of enriched ^64^Ni, other cyclotron-based methods for ^64^Cu production involving proton- and deuteron-irradiation of zinc targets have been investigated. Irradiation of a zinc target with a natural composition with 19 MeV deuterons can produce 33.9 GBq of ^64^Cu via the (d, αn) and (d, 2pxn) reactions. While the product is contaminated with ^61^Cu, high radionuclidic purity can be achieved with a sufficient cooling time.^41^ The use of enriched zinc targets has also been investigated by employing the ^64^Zn(d, 2p) ^64^Cu reaction. Irradiation of a pure ^64^Zn target with 19.5 MeV deuterons does not produce radiogallium impurities and gives a higher yield (31 MBq/μAh), when compared to irradiation of a natural composition target, in the same energy range. However, it also leads to a higher level of ^61^Cu contamination; in addition, ^65^Zn is produced in this route, which can accumulate with the recovery of the target.^61^ In facilities with access to >30 MeV proton beams ^64^Cu can also be obtained in lower yields as a byproduct of ^67^Ga production via the ^68^Zn(p, 2n) ^67^Ga reaction. By this route, ^64^Cu is formed by the ^68^Zn(p, αn) ^64^Cu reaction. For this method to be viable, however, the beam energy must be selected to prevent ^67^Cu formation by the ^68^Zn(p, 2p) channel.^62^ It has been reported that in the energy range 25–10 MeV, a yield of 67 MBq/μAh can be achieved without ^67^Cu contamination. Another possible route is the ^66^Zn(p, 2pn) ^64^Cu reaction, which in the energy range 70–35 MeV produces ^64^Cu with a yield of 777 MBq/μAh; however, the high level of ^61^Cu contamination requires a waiting period, which limits the practical yield to 122 MBq/μAh.^63^

A low-energy route for ^64^Cu production, involving irradiation of a zinc target, is the ^67^Zn(p, α) ^64^Cu reaction. Its cross-section peaks at about 14 MeV, making it practical in medical cyclotrons. The enriched ^67^Zn target is relatively expensive, but it is already used for the production of ^66^,^67^Ga radionuclides.^64^ As ^64^Zn and ^70^Zn are both present, even in the highly enriched [^67^Zn]O target, the main impurities formed are ^61^Cu, produced by the ^64^Zn(p, α) ^61^Cu reaction, and ^67^Cu produced via the ^70^Zn(p, α) ^67^Cu reaction. Those impurities may necessitate longer cooling time before application in the case of ^61^Cu, or limit the application period of labeled compounds in the case of ^67^Cu. However, in the enriched target material, the quantity of ^70^Zn is small as is the cross section of the ^70^Zn(p, α) ^67^Cu reaction in the energy range of medical cyclotrons; therefore, the fraction of ^67^Cu in the product is only a concern for longer irradiation times. As for the ^61^Cu product, its half-life is shorter than that of ^64^Cu, so its quantity in the product decreases during longer irradiations or cooling times after bombardment. This reaction also coproduces gallium radioisotopes, which may complicate the processing and handling of the target, but do not influence the purity of the ^64^Cu. A production test demonstrated, that at saturation conditions, a yield of 479 MBq/μAh can be achieved with 96% purity, rising to 99% 10 h after irradiation.^46^

Cyclotron production of ^64^Cu by activation of a copper target has also been reported. According to the TENDL-2021 database, the ^65^Cu(p, pn) ^64^Cu reaction has a threshold of ∼12 MeV and reaches its maximum at ∼24 MeV, thus making production of ^64^Cu via this route possible in small, medical cyclotrons. Irradiation of an enriched [^65^Cu]O target with a 18.2 ± 0.4 MeV proton beam at saturation conditions does not result in any copper radioisotopic impurities; however, as the target is a stable copper isotope, the specific activity of the formed ^64^Cu (120 MBq/mg) is too low for labeling biological vectors. However, ^64^Cu obtained by this route can be still used as a radiopharmaceutical for PET imaging, in the form of [^64^Cu]Cl_2_. For this purpose, the mass of the target should be minimized to avoid the potential toxicity of the administered copper. The specific activity could also be improved by considering higher beam energies, achievable with research and commercial cyclotrons.^46^

### Isotope ^67^Cu

^67^Cu is the longest-lived copper radioisotope (*T*_1/2_ = 61.83 h), which makes it suitable for use in conjunction with molecules with slow pharmacokinetics, such as antibodies and proteins. It decays by β^–^ decay with mean energy of 141 keV and maximum energy of 562 keV, accompanied by emission of several intense γ lines (91, 93, and 185 keV). The mean energy of emitted β^–^ particles is slightly higher than these of the clinically established ^177^Lu, thus making ^67^Cu a valuable radionuclide for internal radiotherapy. Gamma emission associated with the decay can also be used for SPECT imaging and dosimetric calculations. Additionally it can be paired with its shorter-lived, positron-emitting isotopes for dosimetric calculations and distribution studies.^65−67^

Despite its favorable properties as a promising therapeutic radionuclide, ^67^Cu is characterized by low availability and high cost, and is therefore not as widely studied as ^64^Cu.^65^ Much the same as ^64^Cu, ^67^Cu can be produced in cyclotrons and nuclear reactors via reactions performed on zinc targets.^26^ An important development in its production is the photonuclear reaction, ^68^Zn(γ, p) ^67^Cu, performed using bremsstrahlung photons obtained with linear electron accelerators (LinAcs). As LinAcs are quite common, this method may increase the availability of ^67^Cu around the world.^27^ Recently, thanks to the Department of Energy Isotope Program (DOE-IP), ^67^Cu produced by this method became available in the US in quantities and purities sufficient for medical research.^67^

The photonuclear production of ^67^Cu by the ^68^Zn(γ, p) ^67^Cu reaction possesses several advantages over other production routes. When provided an isotopically enriched target, the reaction does not coproduce ^64^Cu and hence yields ^67^Cu with very high radionuclidic purity.^27^ Enrichment of the target also increases the yield by a factor of ∼5 when compared to irradiation of a natural composition target. Target design and cooling is of great importance, because of the low melting point of zinc.^68^ Second, photonuclear activation produces ^67^Cu with a very high specific activity, suitable for labeling of targeted molecules.^69^ In recent years a large-scale process has been developed in Argonne National Laboratory, in which ^67^Cu is separated from the bulk zinc target by sublimation, thus allowing for rapid processing.^70^ With 40 MeV electron beams with average power of 18.2 kW and a 55.5 g target, 62.9 GBq of ^67^Cu can be achieved in 53.5 h irradiation.^67^

The neutron-induced production of ^67^Cu was investigated in the ^67^Zn(n, p) ^67^Cu and ^68^Zn(n, np) ^67^Cu reactions. ^67^Zn(n, p) ^67^Cu reaction performed using fission neutrons has been investigated for over 50 years.^71^ In this reaction enrichment of the target is of great importance, as ^64^Zn has a higher abundance and the ^64^Zn(n,p) ^64^Cu reaction has a higher cross-section, as such, the bombardment of a natural composition target results in unacceptable level of ^64^Cu contamination; however, irradiation of the enriched ^67^Zn target increases the yield 20-fold and suppresses ^64^Cu production by a factor of 60. Due to the small cross-section of the reaction, high neutron fluxes are also required to achieve satisfactory yields. Coproduction of ^65^Zn is also unavoidable in reactors with a high ratio of thermal neutrons if ^64^Zn is present in the target, which contaminates the recycled target material. Such contamination may be reduced by thermal neutron shielding.

It has been proposed that a fast spectral neutron source may be more effective than a nuclear reactor.^72^ The higher neutron energies obtainable in accelerator-driven neutron generators open up the possibility of ^67^Cu production via the ^68^Zn(n, x) ^67^Cu process, e.g., ^68^Zn(n, d) and ^68^Zn(n, np) reactions, and allow for higher yields in the ^67^Zn(n, p) ^67^Cu reaction. With near 14 MeV neutrons, the ^67^Zn(n, p) ^67^Cu has a cross-section of 43.63 mb. In the energy range of 25–30 MeV, the cross-section of the ^68^Zn(n, x) ^67^Cu reaction is estimated to be over 100 mb, allowing for higher yields.^73^^67^Cu with high radionuclidic purity, obtained by the ^68^Zn(n, np) ^67^Cu reaction, induced by neutrons obtained by irradiating a carbon target with deuterons was used in a biodistribution study in mice.^74^

The ^68^Zn(p, 2p) nuclear reaction is the most widely adopted cyclotron route, as it produces ^67^Cu in high thick target yields, although there are still some discrepancies in the nuclear data.^75^ Its threshold is 10.12 MeV and the cross-section increases with higher incident proton energies, making this production route practical in intermediate energy cyclotrons.^76^ Enrichment of the target increases the yield by four to five times and reduces the coproduction of other radiocopper isotopes. Nevertheless, irradiation of an enriched ^68^Zn target produces a complex mixture of radionuclides, including ^58^Co, ^67^Ga, ^57^Ni, and ^65^Zn, which complicates the consequent purification and target recycling. Additionally, this process yields a large amount of ^64^Cu which contaminates the product and constitutes a major fraction of the produced ^67^Cu, even after a long cooling time.^77^

An alternative to the ^68^Zn(p, 2p) ^67^Cu reaction performed in lower energy cyclotrons is the ^70^Zn(p, α) ^67^Cu reaction. This route has two possible approaches. With proton energies below 14 MeV, it produces ^67^Cu without ^64^Cu contamination, albeit at a much lower yield than the (p, 2p) channel. This drawback can be offset with higher beam currents (>300 μA), achievable in modern cyclotrons, and longer irradiation times.^78^ With higher proton energies, it can produce ^67^Cu with nearly 70% higher yields than the (p,2p) reaction, as the cross section of this reaction is twice as high in this energy range. Although ^64^Cu production is unavoidable at those energies, it can be minimized by leaving an appropriate decay period after bombardment.^79^ However, the two methods can be more expensive than the (p,2p) reaction as they require a highly enriched target: ^70^Zn has natural abundance of 0.61%.^80^ Interestingly, bombardment of a cryogenic hydrogen gas target with a 15 MeV/nucleon ^70^Zn ion beam has been evaluated for the production of ^67^Cu by inverse kinematics. Activities necessary for preclinical studies could be achieved with heavy-ion beams with intensities in the range of particle μA.^81^

Another route of ^67^Cu production using a ^70^Zn target involves bombardment with deuterons via the ^70^Zn(d, x) ^67^Cu reaction. The target should be highly enriched to limit the formation of unwanted side-products.^82^ However, as the ^70^Zn(d, x) ^64^Cu reaction has a threshold of 26.4 MeV while ^67^Cu production has 0 MeV, this channel does not result in ^64^Cu production, assuming appropriate beam energy selection. This route may prove advantageous in future machines producing deuteron currents in the range of mA and with adequate targetry.^83^

The use of alpha beams has also been proposed for ^67^Cu production via the ^64^Ni(α, p) ^67^Cu reaction. Nickel has a higher stopping power for alpha particles, meaning that the reaction requires less expensive enriched target material to produce comparable amounts of ^67^Cu to proton- and deuteron-induced reactions.^84^

### Isotope ^61^Cu

^61^Cu is a positron emitter suitable for PET imaging. Its properties are comparable to ^68^Ga, with the advantages of a lower maximum positron energy (1.216 MeV vs 1.899 MeV) and a more practical half-life (3.33 h vs 68 min).^85^ It is suitable for imaging processes with slow kinetics, e.g., for labeling antibodies and peptides.^86^ Its half-life is also significantly longer than these of ^60^Cu and ^62^Cu, allowing easier off-site production, distribution and processing.^87^ Compared to the better-established ^64^Cu, it has a higher fraction of β^+^ decay, allowing it to be applied in smaller doses, but achieving lower resolution images due to the simultaneous emission of γ rays, like ^60^Cu.^88^

Even though ^61^Cu has better decay characteristics than ^64^Cu,^89^ it has received less attention of the scientific community than its longer-lived counterpart. This may be due to the better-established logistics of production and distribution of ^64^Cu. However, with recent advancements in cyclotron and radiochemistry technology and infrastructure, it is currently regaining attention.

Several methods of ^61^Cu production can be found in the literature. McCarthy et al. obtained significant quantities of ^61^Cu using 14.7 MeV protons and 8.1 MeV deuterons by the ^61^Ni(p, n) ^61^Cu and ^60^Ni(d, n) ^61^Cu reactions. While the proton-induced reaction produced higher yields of ^61^Cu, a highly enriched target was necessary to achieve satisfactory purity, and routine production was too expensive due to the low isotopic abundance of ^61^Ni (0.14%). On the other hand, enriched ^60^Ni target has an advantage of much lower cost, thus making the ^60^Ni(d, n) ^61^Cu route applicable for production of smaller quantities of ^61^Cu.^86^ It is also worth noting, that the deuteron-induced reaction may be performed on a natural composition target, albeit with longer irradiation times.^86^ Svedjehed et al. reported a production method based on ^nat^Ni(d, x) ^61^Cu reaction along with its fully automated purification process.^90^

Another promising approach for ^61^Cu production is the proton irradiation of zinc. The ^64^Zn(p, α) ^61^Cu reaction has been investigated with both natural and enriched targets.^91^ Using a natural target like this is a cheaper and hence more attractive alternative to the proton irradiation of ^61^Ni. Additionally, production of ^61^Cu via this reaction with a liquid target system has also been investigated, which could further increase its applicability for routine production; such approaches offer both reduced processing time and cost, easier recycling of the target material and possibly repurposing of existing infrastructure.^85,89^

Moreover, production of ^61^Cu by α-particle irradiation of natural nickel^92^ and cobalt^93^ has been reported. Natural cobalt includes only one isotope, ^59^Co, which makes it a very appealing target for ^61^Cu production. Because of its low cost, cobalt does not have to be recycled and its radionuclidic purity limits the potential for unwanted side-reactions. However, the use of alpha beams for production of medical isotopes remains low, due to their limited availability.

### Isotopes ^60^Cu and ^62^Cu

^60^Cu has a relatively short half-life of 23.7 min and decays to stable Ni isotopes via β^+^ decay (93%) and electron capture (7%).^94^ The high fraction of positron decay and short half-life make ^60^Cu suitable for PET imaging of processes with fast kinetics. However, the emission of high-energy γ rays in cascade with each positron significantly lowers the image quality by increasing noise and dead time.^86^^60^Cu can be produced efficiently by the ^60^Ni(p, n) ^60^Cu reaction in a biomedical cyclotron, using enriched ^60^Ni as a target.^86^ Its production via ^59^Co(^3^He, 2n) ^60^Cu has also been reported by a multiparticle accelerator,^95^ but such machines are scarce. Generally speaking, the longer half-lives of ^61^Cu and ^64^Cu make them more suitable for off-site production and distribution and ^64^Cu produces higher quality images than those obtained with ^60^Cu.^96^

**Figure 1:**
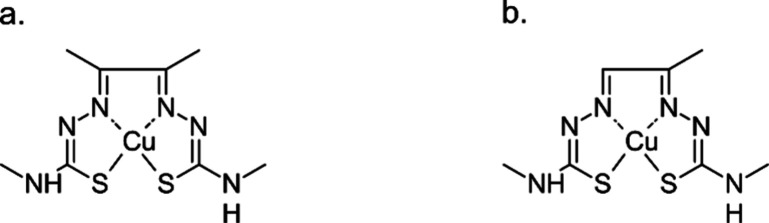
Structures of (a) [Cu]-ATSM and (b) [Cu]-PTSM complexes.

More attention has been devoted to ^62^Cu than ^60^Cu. The fact that is is the shortest-lived medical copper radionuclide (*T*_1/2_ = 9.74 min), and almost exclusively decays by β^+^ decay (98%, EC 2%), make it suitable for PET imaging with small tracer molecules, such as diacetyl bis(*N*(4)-methylthiosemicarbazonato)copper(II) ([Cu]-ATSM, Figure 1a) or pyruvaldehyde bis(*N*(4)-methylthiosemicarbazonato)copper(II) ([Cu]-PTSM, Figure 1b). Its short half-life restricts imaging to less than an hour following injection, but also allows for repeated studies under different conditions.^97^ Importantly, as it is a daughter nuclide of ^62^Zn, it can be produced utilizing a generator system, making it an especially attractive radioisotope for PET facilities without access to a cyclotron.

A radioisotope generator is a system, in which a longer-lived “parent” nuclide continually decays to a shorter-lived “daughter” nuclide. The daughter can be then separated, leaving the parent to regenerate a fresh daughter. There are many examples of such systems, the most prominent being the ^99^ Mo/^99m^Tc generator, which is currently used for routine production of ^99m^Tc in imaging studies.^98^ As they are generally portable and hence can be produced off-site and shipped, and the parent isotope has a longer half-life, they are convenient sources of medical radioisotopes, especially in facilities without access to a cyclotron.

^62^Zn decays by β^+^ decay and electron capture and has a half-life of 9.193 h.^99^ It is usually produced by proton irradiation of natural copper targets in medium energy cyclotrons via the ^63^Cu(p, 2n) ^62^Zn and the ^65^Cu(p, 4n) ^62^Zn reactions.^100−102^ For facilities with access to proton beams with energies above 50 MeV, the ^nat^Zn(p, x) ^62^Zn process was suggested, as it is expected to produce higher yields than the ^nat^Cu+p process in this energy range.^103^

In the 1970s, it was suggested that ^62^Zn and its decay product, ^62^Cu, possess suitable properties for production of ^62^Cu in a ^62^Zn/^62^Cu generator system.^104^ Several designs of such systems were reported. Yagi and Kondo utilized a Dowex 1 × 8 anion-exchange resin with ^62^Zn adsorbed onto it and eluted it with 0.5 M HCl; the process yielded several 100 μCi quantities of ^62^Cu with 70–80% elution efficiency in 10 mL elutions.^105^ Robinson et al. demonstrated a design based on a Dowex 1-X10 anion-exchange resin, eluted with 0.1 N HCl, 10% NaCl solution with 1 μg/mL CuCl_2_ added as a carrier, which reduced the specific activity of the produced ^62^Cu.^106^ This design achieved over 85% recovery of the ^62^Cu in a 3.5 mL volume with less than 0.001% ^62^Zn contamination. A series of improved generator designs were then reported. One, using 25 mm × 5 mm Dowex 1 × 16 200–400 mesh anion-exchange resin, eluted with 1.7 M NaCl and 0.1 M HCl solution, achieved 80–95% of ^62^Cu activity recovery in a volume of 1 mL of eluent.^101^ Another design, based on AG1-x8 anion exchange resin and 0.3 M HCl/40% ethanol eluent, demonstrated >90% elution yield with 3 mL of the eluent and very low amount of ^62^Zn breakthrough (below 3 × 10^–7^%). Direct elution of the generator into a H_2_PTSM kit with a buffer produced a labeled, ready-to-inject radiopharmaceutical in under 5 min, without the need for further processing.^100^ A commercially available microgenerator has recently been developed by Proportional Technologies Inc.^107,108^ It has demonstrated high efficiency and high purity, producing a ready-to-label ^62^Cu solution in 0.25 mL quantities. Additionally, the shielded housing of the microgenerator allows it to be operated without a hot cell. This design has already been successfully tested in a clinical setting.^109^

Another approach to generator design was proposed by Fujibayashi et al.^110^ In this design, the anion-exchange resin was replaced by a CG-120 cation-exchanger with glycine solution as the eluent, capitalizing on differences in complex formation between zinc and copper. The generator showed 70% elution efficiency with >99.9% radionuclidic purity of the produced copper solution. The approach offers the advantages that the produced solution has physiological pH and osmotic pressure and has no added carrier copper. It was also demonstrated that the copper-glycine complex rapidly formed a complex with bis(thiosemicarbazone) (BTSC) by simple ligand exchange reaction. However, the potential use of this design was limited by radiation safety concerns due to a high amount of ^62^Zn breakthrough through the column (2.2%), and the high cost of the parent nuclide production. The addition of a Sep-Pak Plus CM Cartridge increased elution efficiency to ∼96% and resulted in very low ^62^Zn breakthrough, i.e., less than 0.1% on first elution. Large-scale production of this generator has been established in Japan and the clinical application of the produced ATSM labeled with ^62^Cu has been approved by National Institute of Radiological Sciences (NIRS).^102^

Nevertheless, ^62^Zn/^62^Cu generators have their limitations. Due to the short half-life of the ^62^Zn parent, they have a short shelf life of one to 2 days. This downside is however offset by the ability to produce radiopharmaceutical doses for several patients as generator production can be scaled in response to demand.^109^

Direct production of ^62^Cu by 62Ni(p, n) ^62^Cu^111^ and ^59^Co(α, n) ^62^Cu^112^ reactions has also been reported. Although proton irradiation of a nickel target gives a very high yield, the reaction has been seldom used as an expensive enriched target is required to achieve satisfactory radionuclidic purity. The ^59^Co(α, n) ^62^Cu reaction could be performed in a multiparticle accelerator facility, but the availability of such beams restricts its widespread adoption.

### Chemistry of Copper Radiopharmaceuticals

The use of copper in radiopharmaceutical applications is mostly limited to its (II) oxidation state. Although copper also exists in other oxidation states, such as Cu(I) and Cu(III), Cu(I) complexes are typically too labile for use as radiopharmaceuticals^113^ and complexes of Cu(III) are often thermally unstable and hard to isolate.^114^ Cu(II) has a d^9^ electron configuration and is classified as a borderline hardness cation, which makes it favor nitrogen- and oxygen- donors. It commonly adopts a tetragonal geometry with four shorter equatorial bonds and one or two longer axial bonds, but other conformations are also known.^22,25,115^

In most targeted radiometal-based radiopharmaceuticals, the metal is bound to a pharmacophore by a BFCA, which forms a stable covalent linkage between the label and the targeting ligand (vector) and ensures the stable complexation of the metal *in vivo*. In many instances, the chelator and the vector are connected via a spacer moiety to separate the individual components of the conjugate in order to avoid potential interference. Chelators contain several functional groups for coordination to the radiometal of choice. Radiometal complexation with chelator-modified vectors offers convenient access to radiopharmaceuticals, e.g., by enabling kit formulations.^17,109,116,117^

Radiopharmaceuticals containing copper typically can be into two major groups. One of them comprises small coordination complexes, while the other includes macrocyclic chelator complexes conjugated to a target moiety, such as peptides, proteins and nanoparticles.^118^ Of course, there are also compounds, that escape this narrow classification: for example, some small coordination complexes can be used as bifunctional ligands, linked to a vector. As copper is an important trace element, it can also be used “as is”, in the form of simple inorganic molecules, such as [^64^Cu]Cl_2_.^119^

In metal complex radiopharmaceutical design, the stability of the complex is of great importance, as dissociation of radiocopper from the complex can lead to nonspecific organ uptake and introduction of a background signal, decreasing the signal-to-noise ratio in imaging.^120^ Thermodynamic stability is characterized by the constant of complex formation (*K*_ML_), with a log *K*_ML_ score over 18 typically indicating a more thermodynamically stable complex. In contrast, kinetic stability is characterized by the rate constant of complex dissociation (*k*_reverse_); *in vivo*, this value serves as the key factor determining the clinical applicability of a radiopharmaceutical due to its low concentration in the body^121^ There are several biomolecules present in the human body that possess affinity for copper, with these being extracellular (serum albumin, ceruloplasmin, transcuprin) and intracellular (transporters, chaperones, metallothioneins, superoxide dismutase, cytochrome c),^122^ thus, a viable chelator needs to be sufficiently inert to transchelation by those molecules.^123^

It is also important not to overlook the conditions under which the complex is formed (e.g., reaction time and temperature). Long reaction times are not suitable for radionuclides with short half-lives, and the temperature at which the complex is formed is also important, as most targeted radiopharmaceuticals in development are conjugates with biomolecules. Furthermore, excessively harsh chelating conditions may denature the vector molecules and thus limit their biological activity. Ideally the complex should be formed in less than 15 min at a mild reaction temperature.^121^

Finally, the pharmacokinetics of the chelator can be influenced by various other factors, such as the charge, hydrophilicity and size of the complex. It has been demonstrated that vectors exhibit different biodistribution patterns when conjugated to different chelators but labeled with the same copper isotope.^124−126^ As such, the determination of the best copper chelator for radiopharmaceutical applications is profoundly difficult.

### Acyclic Copper Chelators

**Figure 2:**
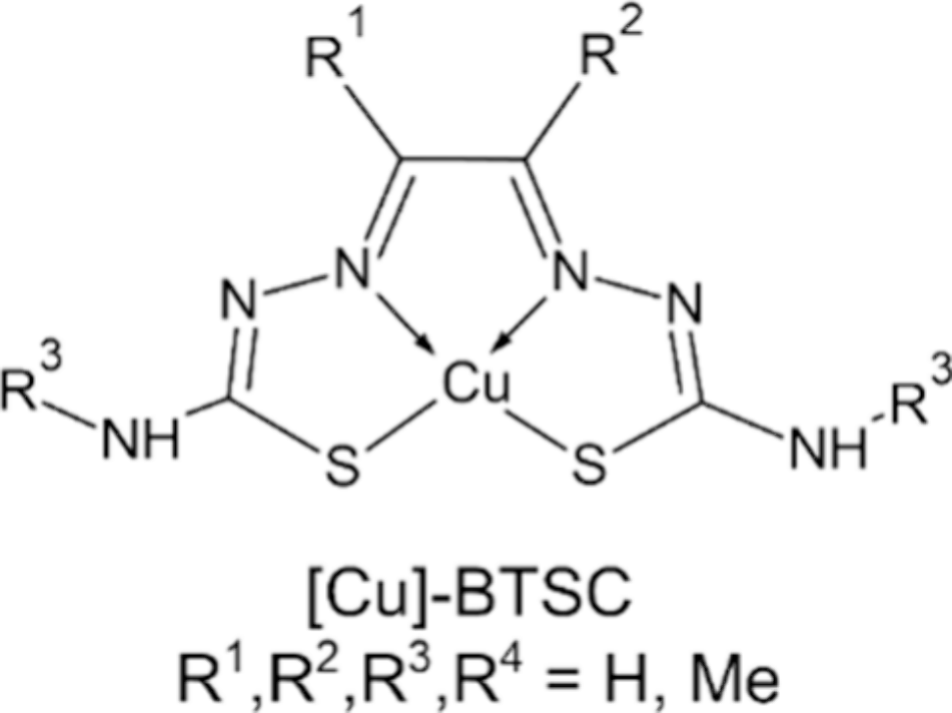
Cu-BTSC general structure.

The most important acyclic copper chelators are BTSC, these being N_2_S_2_-type semicarbazone derivatives. [Cu]-BTSC (Figure 2) complexes are planar, neutral, stable and suitably lipophilic to medical purposes. The radiolabeling process can be performed at approximately physiological pH values, and occurs instantly at room temperature, which is great advantage for practical kit formulations.^127^ BTSC molecules can be also modified, especially in the R^1^ and R^2^ groups attached to the imine backbone. These molecule fragments can affect the Cu^2+^/Cu^+^ redox potential, the release of copper from the complex, the ability to permeation through cell membrane and the antineoplastic activity.^54^ The first Cu-BTSC derivative successfully used in medicine was ^62^Cu labeled diacetyl-bis(*N*4-methylthiosemicarbazone) ([^62^Cu]-ATSM).^128^

### Macrocyclic Copper Chelators

**Figure 3:**
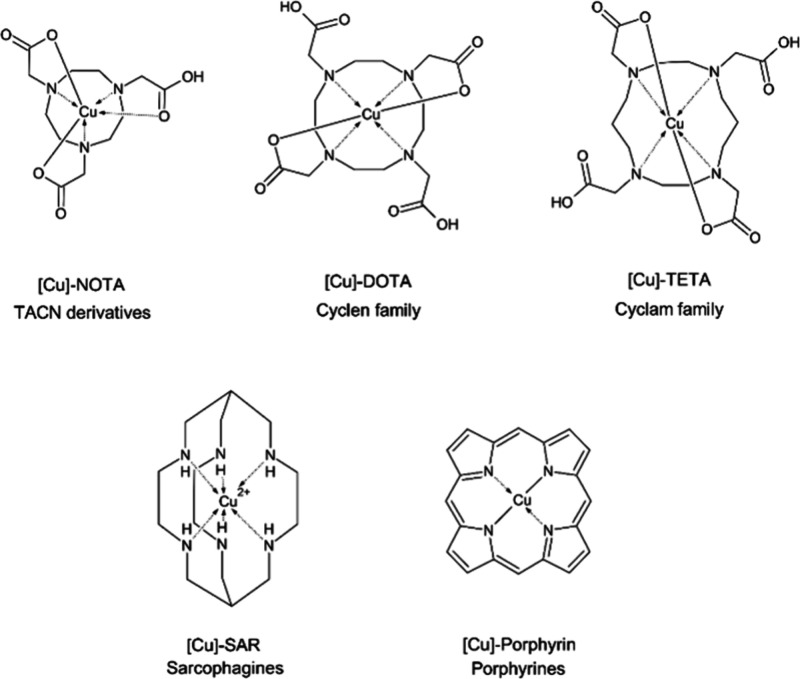
Most common general examples of macrocyclic copper chelators.

The macrocyclic chelators comprise a large family of compounds (Figure 3). These are more commonly used in copper radiopharmaceuticals than acyclic moieties because of their higher stability, which is dependent on the chelate ring size and the cavity size. It has been found that 1,4,7-triazacyclononane (TACN) derivatives such as 2,2′,2″-(1,4,7-triazacyclononane-1,4,7-triyl)triacetic acid (NOTA) have a very small cavity for copper ions, leading to a distorted trigonal prismatic geometry around the copper ion. However, the NOTA ligand is able to quickly protonate the complex, and increase its copper affinity, thus facilitating fast complexation (milliseconds) even at room temperature, leading to stable complex formation (log *K*_ML_ = 23.33) even at low pH.^54,129^

In addition, in 12-aneN_4_ compounds such as cyclen family members, e.g., 2,2′,2″,2‴-(1,4,7,10-tetraazacyclododecane-1,4,7,10-tetrayl)tetraacetic acid (DOTA), the cavity is too small for a copper ion; as a result, the metal atom is slightly outside the plane of the macrocyclic ring. Therefore, the complex adopts a pseudotetrahedral or distorted octahedral geometry, which affects its stability constant (log *K*_ML_ = 24.8). As a result, cyclen family moieties complex copper ions more slowly than TACN derivatives and require high temperatures to complete the process.^54,129,130^

In the cyclam family (14-aneN_4_), e.g., 1,4,8,11-tetraazacyclotetradecane-1,4,8,11-tetraacetic acid (TETA), the compounds have sufficient cavity size to locate in-plane copper inside the ring, which results in more stable complexes (log *K*_ML_ = 28.9), Unfortunately, cyclam family moieties are characterized by relatively high copper dissociation *in vivo*. In response, cross-bridged cyclam derivatives, which are significantly more stable under physiological conditions, have been developed.^54,131,132^

Another group of macrocyclic compounds are the sarcophagines, commonly knowns as SARs. They are capsule-shaped heaxamine-macrobicyclic cage amine ligands. SARs are characterized by very high kinetic stability as copper complexes and protect the complexed copper ion against reduction and *in vivo* transchelation. Moreover, radiolabeling processes with SARs can proceed across a wide pH range (pH 4–9) in a few minutes at room temperature with even 100% yield.^28,54,133,134^

Porphyrins are large macrocyclic moieties with a porphyrin core structure. Despite being the objects of increasing interest in radiocopper science, they are not yet as widely studied as the other derivatives discussed above. This family of chelators is characterized by a cavity size sufficient to complex copper ion at room temperature, thus forming planar complexes that are stable under physiological conditions, but the chelator moiety is large enough to allow the family to conjugate exclusively with large ligands, e.g., peptides, proteins or nanoparticles.^54,135^ Moreover porphyrins can be accumulated in tumor tissue in many ways, which further increases the uptake of radiocopper by tumor cells, and positively influences its therapeutic or diagnostic effect. It has been reported that porphyrins can interact with low-density lipoproteins, which may lead to the internalization of the lipoproteins and the subsequent intracellular release of radiolabeled porphyrins.^136^ Another mechanism involves tumor-associated macrophages, which are responsible for growth factor production and angiogenesis promotion, as well as reduction of tumor growth or destruction of neoplastic cells. These macrophages can phagocytose protein-conjugated porphyrins or large porphyrin aggregates, which increases the overall tissue uptake of radioactive copper.^135,137^

## Selected Advances in the Development of Copper Radiopharmaceuticals

### Alzheimer’s Disease

AD is a neurodegenerative disease causing cognitive decline. During its course, extracellular aggregates of amyloid-β (Aβ) are formed. The main alloforms of Aβ are Aβ_40_ and Aβ_42_, of which, Aβ_40_ is present in the deposits in larger amounts, but Aβ_42_ exhibits higher neurotoxicity and aggregates more easily. The neurotoxicity of soluble Aβ oligomers is related to their interactions with cell membranes and synaptic receptors, influencing intracellular systems and neurotransmission. Amyloid deposits were also found to contain uncommonly high concentrations of metal ions, such as Fe^2+^, Cu^2+^, and Zn^2+^, which promote the formation of Aβ aggregates and cause the formation of reactive oxygen species (ROS) through Fenton-like reactions. Thus, small molecules inhibiting the interaction between metal ions and Aβ peptides have potential to be used as therapeutic compounds. Additionally, PET tracers with affinity for amyloid plaques could be used for early assessment of the disease and to monitor its progress. Emerging PET tracers binding to Aβ include florbetapir, flumetamol, florbetaben and Pittsburgh Compound B. All of these compounds are aromatic hydrophobic molecules, binding noncovalently to the hydrophobic pockets and channels formed in the filaments by the parallel orientation of β sheet structures. However, ^11^C and ^18^F have short half-lives and their incorporation into tracer molecules requires organic synthesis; as such, radiopharmaceuticals based on metals such as ^64^Cu and ^61^Cu are attractive alternatives. Metal-based compounds can be made into “kit formulations”, which combined with relatively long half-lives of ^61^Cu and ^64^Cu, makes their logistics more approachable.^138,139^

**Figure 4:**
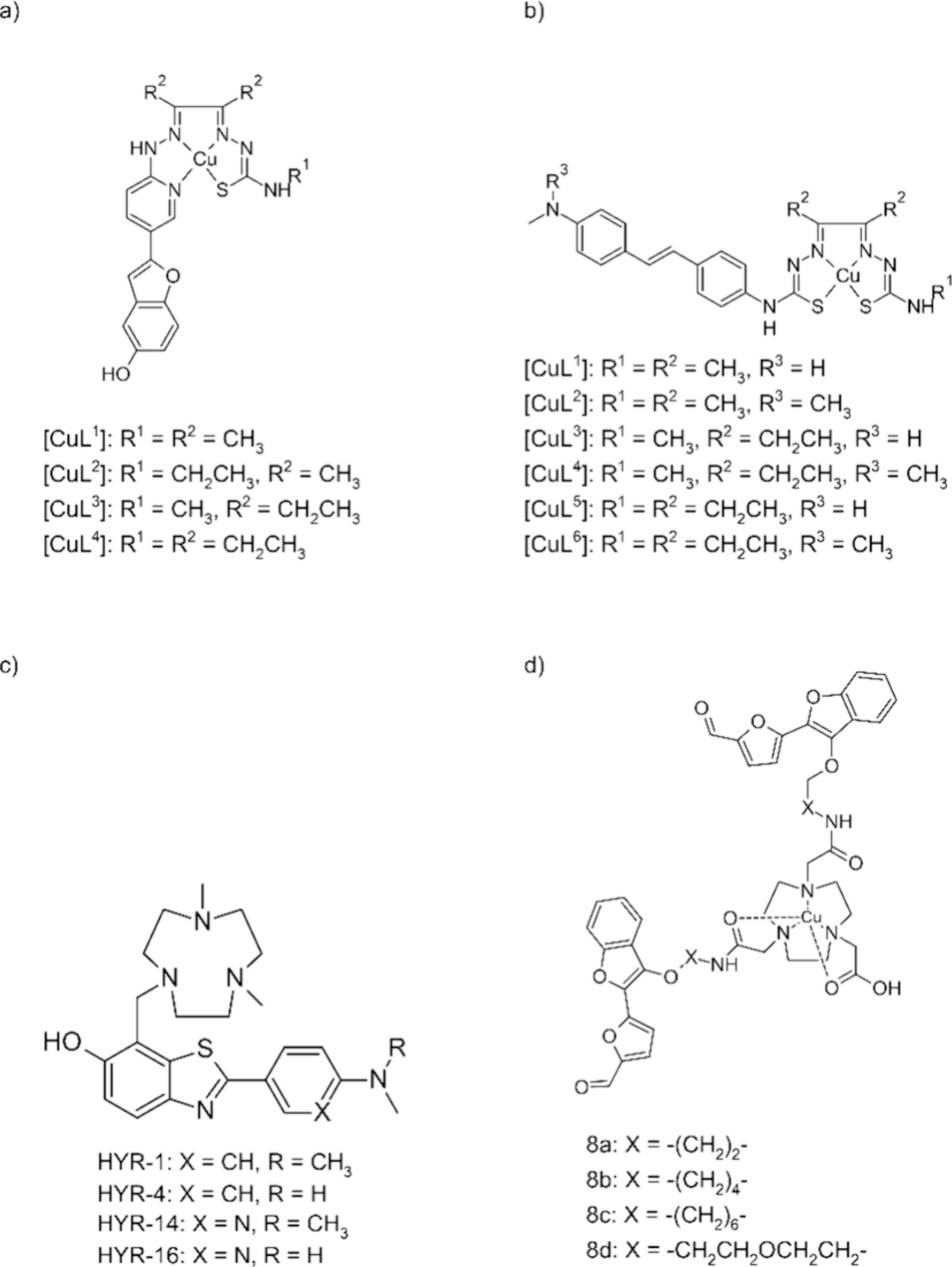
Compounds investigated as potential PET tracers for Alzheimer’s Disease: (a) Hybrid thiosemicarbazone-benzofuran copper complexes. (b) Copper complexes of bis(thiosemicarbazone) with appended stilbenyl group. (c) HYR family of compounds bearing amyloid-beta binding moiety inspired by the structure of Pittsburgh Compound B combined with triazacyclononane metal chelating group. (d) Copper complexes of divalent ligands comprising 1,4,7-triazacyclononane-1,4,7-triacetic acid (NOTA) chelator bound to two 2-(2-formyl-5-furanyl)-3-hydroxymethylbenzofuran moieties.

A 2018 study examined a series of four hybrid thiosemicarbazonato-benzofuran ligands that could be used as PET tracers labeled with copper radionuclides (Figure 4a). The design was inspired by the low nonspecific binding of another potential PET tracer, [^18^F]NAV4694; it was found that derivatives of 2-pirydylbenzofuran bind to Aβ plaques with higher specificity than styrylpiridine and benzothiazole derivatives, with less white matter binding. All reported molecules rapidly formed charge-neutral copper(II) complexes at room temperature. In addition, they were found to be stable, as indicated by conditional metal ion stability constants of 10^–18^ M against ethylenediaminetetraacetic acid (EDTA) (at pH 7.4) and resistance against competition challenges by Na_2_H_2_EDTA and human serum albumin. They were also harder to reduce to copper(I) and subsequently remove the metal ion than [Cu]-ATSM, as indicated by cyclic voltammetry. In biodistribution studies in C57BI/6 mice, the highest metabolic stability among the studied compounds was noted for the CuL_4_ complex, bearing ethyl groups on the BTSC framework: it demonstrated a *t*_1/2_ of 29 min in human liver microsomes and 21 min in mice, and exhibited the highest brain uptake at 2 min postinjection, with fast clearance from both the brain and the liver. CuL_4_ also preferentially binds to areas of the brain rich in Aβ, with improved differentiation between white and gray matter, as identified using laser ablation-inductively coupled plasma mass spectrometry in post-mortem human brain tissue. It was thus determined that CuL_4_ radiolabeled with any of the positron-emitting copper isotopes is a promising agent for the assessment of amyloid pathology in AD patients.^138^

A 2020 study examined a group of ligands, based on the bis(thiosemicarbazone) framework, incorporating a stilbenyl moiety. To improve on previous work, rather than attaching the Aβ-binding group by a hydrazone bond, which is prone to hydrolysis, the group was directly attached to the fourth-position nitrogen in the thiosemicarbazone scaffolding by a transamination reaction between *N*4-methyl-*N*4-phenyl-3-thiosemicarbazone and amino-stilbene. The stilbene group was appended with electron-donating methylamino or dimethylamino substituents to allow the complex to bind more effectively to Aβ plaques. *In vitro* studies suggest, that the complexes are resistant to reduction and consequent dissociation in the presence of glutathione, can be labeled with ^64^Cu at room temperature, have appropriate lipophilicity for blood-brain barrier (BBB) permeation and interact with Aβ_1–40_ peptides.^140^ Two of the complexes with low molecular weight were selected for further investigation, as this would them more suitable for BBB penetration. Both were found to selectively bind to Aβ plaques without significant retention in normal brain tissue, as indicated by epi-fluorescence microscopy in post-mortem brain tissue from AD patients and control non-AD tissue. Biodistribution studies in wild-type Balb/c mice indicated that the complexes penetrate the BBB to a degree similar to that of [^64^Cu]-ATSM and exhibit good clearance at 60 min postinjection. As such, CuL^2^ and CuL^6^ (Figure 4b) were identified as most promising for future work.^140^

Another study, inspired by Pittsburgh Compound B, examined a series of multifunctional compounds combining a 2-aryl-benzothiazole Aβ binding moiety with a 1,4,7-triazacyclonane metal-chelating group (Figure 4c). The described compounds could be either used as disease-modifying drugs which suppress the interactions between metal ions and Aβ peptides or could be labeled with positron-emitting copper radionuclide and used as potential imaging probes. Biodistribution studies on normal CD-1 mice found ^64^Cu-labeled HYR-17 to demonstrate a significant brain uptake up to 0.99 + −0.04% of the injected dose per gram. Notably, HYR-16 exhibited most favorable properties for alleviation of neurotoxicity of soluble Aβ oligomers. All of the compounds can be radiolabeled, but HYR-4, HYR-17, and HYR-18 also demonstrated acceptable lipophilicity and specific radiolabeling of amyloid plaques, as indicated by D_oct_ measurements and *ex vivo* autoradiography studies in brain sections of 5xFAD mice. Based on these characteristics, the HYR-16 has potential for further investigation in the treatment of AD while HYR-17 radiolabeled with ^64^Cu may be used in PET imaging of amyloid plaques.^139^

A different approach for the design of Aβ-binding ^64^Cu-based tracers was also reported. A 2020 study examined multivalent compounds composed of NOTA chelator bound to two 2-(2-formyl-5-furanyl)-3-hydroxymethylbenzofuran moieties via ethyl, butyl, hexyl or ethylene glycol spacers intended to optimize the lipophilicity of the compounds. 2-(2-formyl-5-furanyl)-3-hydroxymethylbenzofuran earlier demonstrated binding to soluble Aβ species *in vitro* and *in vivo*. *Ex vivo* autoradiography studies revealed that divalent complexes exhibited higher signal intensities compared with their monovalent analogues. *In vitro* and biodistribution studies in mice demonstrated that the compounds selectively bind to amyloid plaques and cross the BBB. Two compounds exhibiting the most favorable properties were then selected for positron emission tomography combined with computed tomography (PET-CT) imaging studies in wild-type and 5xFAD mice. The 8b (Figure 4d) compound, containing a butyl spacer moiety, was found to demonstrate the highest potential as a PET tracer for AD.^141^

### Breast Cancer

#### Bombesin

The gastrin-releasing peptide receptor (GRPR) is strongly presented on the surface of various cancer cells, particularly breast, colon, lung, pancreas, and prostate cancer. In contrast, the expression of GRPR is generally low in healthy tissues, except in the pancreas.^142^

**Figure 5:**
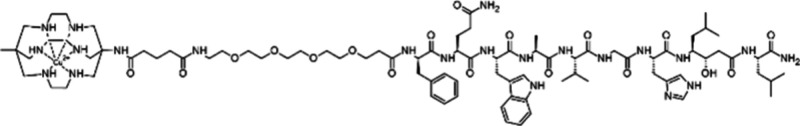
Structure of [^64^Cu/^67^Cu]-SAR-BBN complex.

A 2022 paper discusses a clinical phase I study that evaluated the efficacy of [^64^Cu]-sarcophagine-bombesin (Figure 5) PET-CT as a diagnostic agent for restaging metastatic ER+/PR+/HER2– breast cancer. Bombesin (BBN) is a tetra-decapeptide that demonstrates a strong preference for GRPR, and its absorption in breast cancer has been confirmed in clinical studies involving humans. In the study, BBN was bound with the chelator SAR and ultimately labeled with copper-64. The study population comprised patients who demonstrated reduced sensitivity to ^18^F-fluorodeoxyglucose PET-CT (FDG). The trial findings suggest that [^64^Cu]Cu-SAR-BBN PET-CT is a safe imaging technique with potential diagnostic significance in metastatic ER+/PR+/HER2– breast cancer, specifically in the lobular subtype. However, additional examination is necessary.^143^

#### Trastuzumab

The HER2 receptor plays a significant role in promoting tumor growth in 20% of breast cancers. The first targeted treatment for HER2+ breast cancer to receive FDA approval was the humanized monoclonal antibody trastuzumab, which inhibits the growth of cultured HER2+ breast cancer cells by binding to the extracellular domain of HER2.^144^ Trastuzumab was combined with DOTA and radiolabeled with copper-64. This compound has been studied for its potential application in the theranostics of breast cancer.^145,146^ A 2018 study confirmed the potential application of [^64^Cu]-DOTA-trastuzumab PET-CT in modifying therapy for HER2+ patients with metastatic breast cancer: the findings indicate a direct relationship between the absorption of [^64^Cu]-DOTA-trastuzumab by the tumor and the HER2 status. Since this time, [^64^Cu]-DOTA-trastuzumab has been used to forecast the reaction to specific treatment.^147^

In 2022, a study of 10 women diagnosed with metastatic HER2+ breast cancer examined whether [^64^Cu]-DOTA-trastuzumab PET-CT could predict the response to trastuzumab-emtansine (T-DM1), an antibody-drug conjugate combining trastuzumab with the potent cytotoxin DM1.^148^ Their conjugation is achieved via a noncleavable maleimidomethyl cyclohexane-1-carboxylate (MCC) thioether linker. DM1 is derived from the maytansinoid toxin, which induces cell death by blocking the process of tubulin polymerization. Although DM1 has a narrow therapeutic window for treating cancer, this is expanded by its connection to trastuzumab, which specifically directs the cytotoxin to cancerous cells that have an excessive amount of the HER2 receptor tyrosine kinase. T-DM1 received approval by the European Medicines Agency and FDA in 2013 for the treatment of HER2-positive metastatic breast cancer.^149^

In the study, [^64^Cu]-DOTA-trastuzumab PET-CT was used to quantify tumor uptake, and ^18^F-FDG PET-CT to evaluate tumor response to T-DM1. [^18^F]-FDG PET-CT is a routinely used tracer for imaging tumors and predicting therapy response. The uptake of [^64^Cu]-DOTA-trastuzumab in the tumor was quantified by measuring the maximum standardized uptake value (SUV_max_) 1–2 days after injection. People who responded to TDM1 treatment demonstrated greater tumor uptake compared to those who did not. The results indicate that using [^64^Cu]-DOTA-trastuzumab PET-CT to monitor the absorption of trastuzumab in tumors shows promise in identifying patients with metastatic breast cancer who will benefit from T-DM1 treatment^148^

In another trial, pretreatment magnetic resonance imaging (MRI) and [^64^Cu]-DOTA-trastuzumab PET-CT was used to predict the surgical response to a combination of cytotoxic chemotherapy and the antibodies trastuzumab and pertuzumab in women with HER2-positive breast cancer. Pertuzumab is an anti-HER2 antibody that has been demonstrated to substantially enhance the rates of invasive-disease–free survival in patients with HER2-positive early breast cancer when combined with chemotherapy and trastuzumab.^150^ The biophysical mathematical model utilizes quantitative data obtained from MRI and PET scans to estimate tumor density, perfusion, and HER2+ targeted antibody distribution for individual patients. The model provides an anticipated treatment response for both cases with HER2-targeted therapy and those without.^151^ A clinical study evaluating the use of [^64^Cu]-DOTA-trastuzumab in PET for women with advanced HER2-positive breast cancer is at its final stage. (clinical trial ID NCT01093612).

In addition to DOTA, Trastuzumab can be effectively labeled with ^64^Cu using NOTA. Research indicates that [^64^Cu]-NOTA-trastuzumab is a viable PET imaging agent for HER2+ breast cancer. This compound can be employed to identify candidates and optimal period for HER2 therapy, assess the effectiveness of HER2-targeted treatment, and detect the spread of cancer to distant or metastatic sites. Moreover, [^64^Cu]-NOTA-trastuzumab demonstrated lower absorption than [^64^Cu]-DOTA-trastuzumab in the heart, liver, and spleen. This suggests that [^64^Cu]-NOTA-trastuzumab releases less ^64^Cu than [^64^Cu]-DOTA-trastuzumab, making [^64^Cu]-NOTA-trastuzumab more stable.^152^ A later study validating the safety and feasibility of [^64^Cu]-NOTA-trastuzumab found it to be free of side effects and to offer a comparatively low level of radiation exposure.^153^

### Wilson’s Disease

Wilson’s disease (WD) is a hereditary condition characterized by a mutation in the ATP7B gene and a resulting malfunctioning of its protein. This disorder causes a buildup of copper in many organs, particularly the liver and brain, leading to hepatic and neurological symptoms.^154^

In 2022, a group of researchers conducted a clinical investigation to measure the rate at which copper is metabolized in the liver of patients with WD, as compared to heterozygotes and a control group. Imaging was performed using ^64^Cu PET, as it exhibits exceptional spatial and temporal resolution, making it well-suited for tracking the movement of copper between the blood, liver, bile, and other compartments with a sluggish rate of turnover.^154^ The effect of ^64^Cu as a tracer in WD has previously been confirmed in animal studies.^155^ The findings confirm that ^64^Cu PET imaging allows for the visualization and measurement of hepatic copper retention in WD.^154^

Another study investigates the distribution and elimination of copper in a mouse model of WD after therapy with VTX-801, using [^64^Cu]Cl_2_. VTX-801 is a hepatotropic recombinant adeno-associated vector containing an ATP7B minigene. The administration of VTX-801 to WD mice led to a notable decrease in the amount of ^64^Cu that accumulated in the liver, the recovery of copper excretion through feces, and the normalization of copper’s movement in the bloodstream. Evidence demonstrates that the introduction of VTX-801 effectively corrects copper metabolism in a mouse model of WD over an extended period of time. Moreover, [^64^Cu]Cl_2_ demonstrated efficacy as a viable agent for monitoring the therapeutic impact of VTX-801 in the future clinical trial.^156^

In addition to copper accumulation, WD is linked to a higher concentration of nonceruloplasmin-bound copper in the bloodstream. A crucial goal in the treatment of WD is in fact to ensure that the level of copper not bound to ceruloplasmin remains within the normal range, as this unbound copper is believed to be harmful to the liver and brain. A recent study found that PET-CT scans using [^64^Cu]Cl_2_ can be applied to visualize the distribution kinetics of unbound copper. The study assessed the distribution of nonceruloplasmin-bound copper within 90 min after an intravenous injection in patients with WD, as well as healthy individuals, and healthy individuals carrying a disease-causing ATP7B mutation.^157^

### Prostate Cancer

#### Bombesin

In addition to breast cancer, SAR-BBN PET-CT has the potential to be exploited in the theranostic management of prostate cancer, due to its high expression of GRPR. A study evaluated the use of [^67^Cu]-SAR-BBN treatment in mice with tumors from the PC-3 cell line (Figure 5). The treated group exhibited both greater suppression of tumor growth and extended median survival compared to the control group, indicating that ^67^Cu-labeled SAR-BBN is effective in an animal model and that [^64^Cu]-SAR-BBN demonstrates good potential as a theranostic pair in malignancies expressing GRPR.^142^

#### Ligands Targeting Prostate Specific Membrane Antigen

PSMA, or prostate specific membrane antigen, is a carboxypeptidase that is found on the cell membrane and is highly expressed in prostate cancer. Radiolabeled peptidomimetic inhibitors of PSMA and those possessing a lysine-ureido-glutamate functional group are considered to be efficient tracers for visualizing prostate cancer with PET imaging.^158^

**Figure 6:**
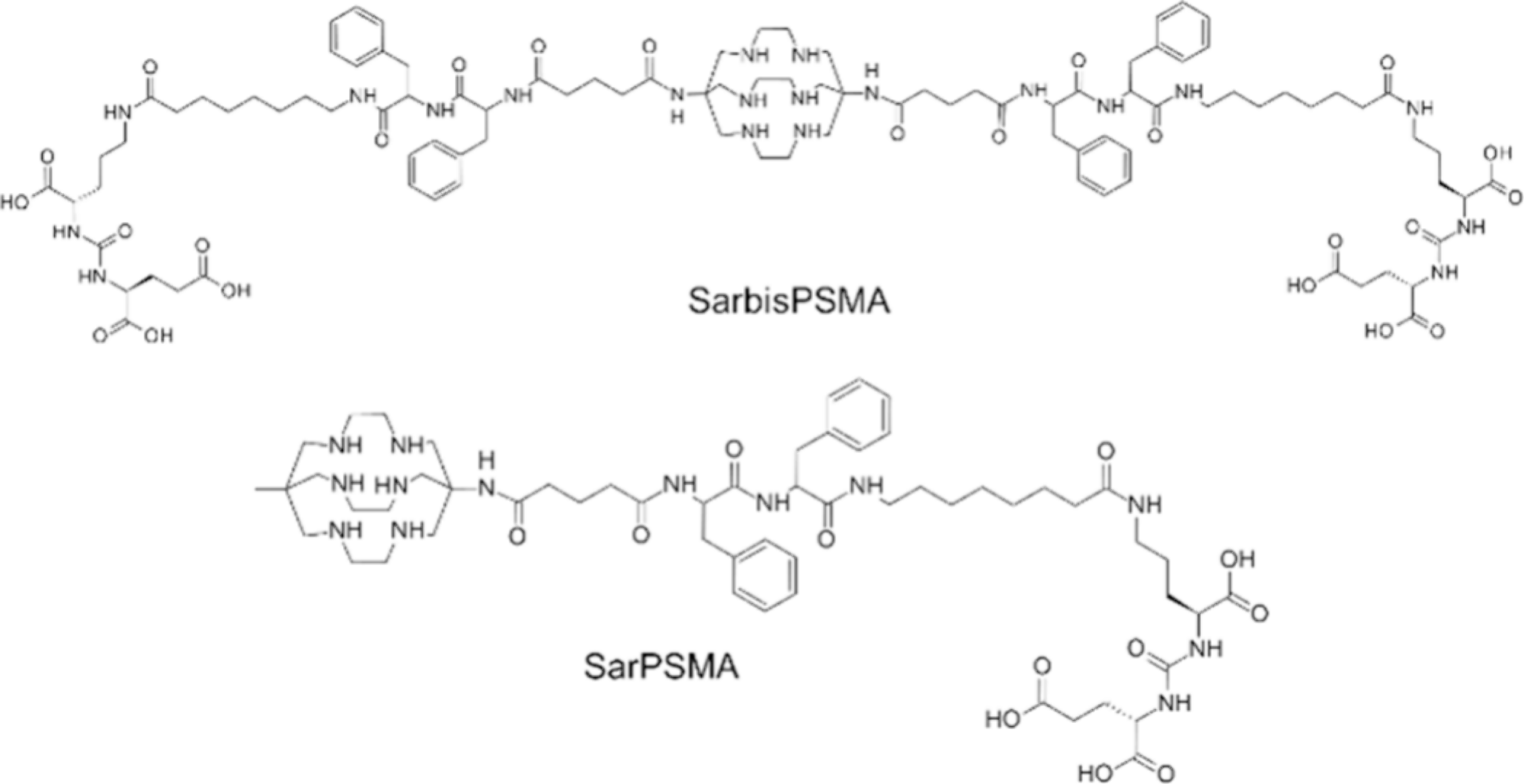
Structures of Sar ligands bearing PSMA functional groups: SarbisPSMA and SarPSMA.

Macrobicyclic SAR ligands, which are attached to either one (SarPSMA) or two (SarbisPSMA) lysine-ureido-glutamate functional groups, have been synthesized and radiolabeled with ^64^Cu (Figure 6). The produced compounds selectively bind to PSMA. In 2019, the properties of both tracers were assessed in NSG mice xenografted with PSMA-positive LNCaP (human prostate adenocarcinoma) tumors. The study demonstrated that [^64^Cu]-SarbisPSMA has significantly greater tumor absorption and retention compared to the monomeric molecule. Nevertheless, [^64^Cu]-SarPSMA exhibits comparable tumor uptake to [^68^Ga]-PSMA-11, a PSMA imaging agent commonly employed in clinical settings. Based on these findings, [^64^Cu]-SarbisPSMA has the potential for use in the diagnosis of prostate cancer. In addition, it could be employed to conduct additional dose measurements for the therapeutic application of a ^67^Cu analogue.^159^

A 2021 investigation assessed the therapeutic effectiveness of a bivalent agent labeled with ^67^Cu–[^67^Cu]-SarbisPSMA. An identical PSMA-positive tumor model was utilized. Xenograft growth was assessed following treatment with [^67^Cu]-SarbisPSMA or [^177^Lu]-PSMA for imaging and therapy. [^177^Lu]-PSMA is an FDA-approved tracer for treating prostate cancer. ^177^Lu and ^67^Cu have comparable β emission energy levels. The study demonstrated that tumor growth was equally suppressed by both indicators in a dose-dependent manner. The shorter half-life of ^67^Cu (61.9 h) compared to ^177^Lu (6.7 days) allows for more frequent dosing within a shorter period, which could enhance the management of fast-growing tumors. Two rounds of 15 MBq of activity reduced tumor growth as effectively as a single 30 MBq treatment. Future research could explore the effectiveness of giving four cycles of 7.5 MBq of activity.^158^ A clinical trial is currently being conducted to evaluate both the safety and effectiveness of [^64^Cu]-SarbisPSMA and [^67^Cu]-SarbisPSMA as a theranostic agent in PSMA-expressing metastatic castrate-resistant prostate cancer (clinical trial ID NCT04868604).

Bifunctionalized SAR chelators are able to create extremely stable complexes with ^64^Cu^2+^ when attached to peptides or peptide dimers. MeCOSar, i.e., 5-(8-methyl-3,6,10,13,16,19-hexaaza-bicyclo[6.6.6]icosan-1-ylamino)-5-oxopentanoic acid, is a bifunctional compound derived from Sarcophagine. It was combined with a PSMA-targeting molecule, i.e., *N*ε-(2-(4-iodophenyl)acetyl)lysine albumin binding group, by a reaction between an *N*-hydroxysuccinimydyl (NHS) ester derivative of MeCOSar and the *N*ε of Lys. Hereby a ligand known as RPS-085 was produced that can be efficiently radiolabeled both with ^64^Cu and ^67^Cu at low concentrations.^160^

**Figure 7:**
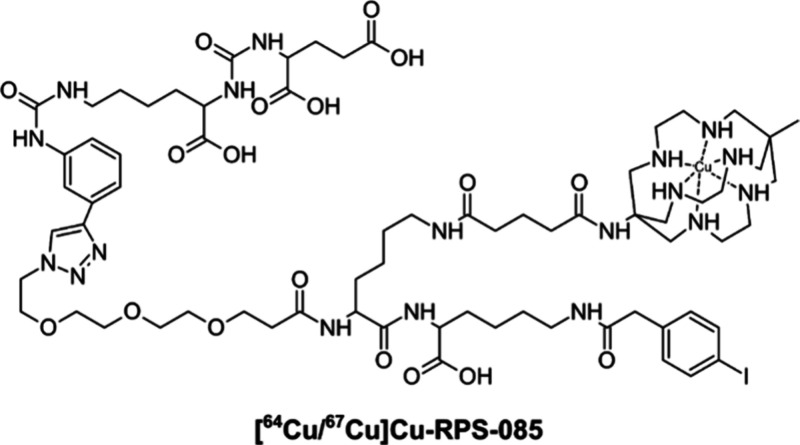
Structure of [^64^Cu/^67^Cu]-RPS-085 complex.

A 2020 report examined the synthesis of [^64^Cu/^67^Cu]-RPS-085 (Figure 7) and its preclinical analysis for prostate cancer PET imaging and treatment. The distribution of [^64^Cu/^67^Cu]-RPS-085 was studied after injection into male BALB/c mice with LNCaP tumor xenografts. The tumors were readily visible with [^64^Cu]-RPS-085 because of its rapid tissue distribution and clearance. Furthermore, both [^64^Cu]-RPS-085 and [^67^Cu]-RPS-085 demonstrate significant absorption in tumors and impressive ratios between tumor and surrounding tissue, even shortly after administration. The therapeutic range of [^67^Cu]-RPS-085 may be similar to that of [^177^Lu]-PSMA-617. As such, [^64^Cu/^67^Cu]-RPS-085 demonstrates significant potential as PSMA-targeting theranostic ligands for imaging and treating prostate cancer.^160^

#### Copper-64 Chloride

^64^Cu may be used in theranostics of tumors with high hCtr1 transporter expression, such as prostate cancer, bladder cancer, hepatocellular carcinoma, glioblastoma multiforme, malignant melanoma, and lung cancer^119^

In a clinical study performed on six healthy participants, uptake of ^64^Cu after intravenous administration was observed in tissues demonstrating high expression of the CTR1 transporter, such as liver, pancreas, intestinal walls and kidneys. After oral administration, ^64^Cu was found to be rapidly absorbed from the gastrointestinal tract and deposited primarily in the liver. ^64^Cu was not excreted in urine and only a negligible amount was detected in feces during the 20 h observation period. Both intravenous and oral administrations of 50 MBq ^64^Cu were sufficient for sequential studies in humans, yielding high quality images up to 20 h after administration.^161^

A prospective clinical study (NCT04703543) examined the efficacy of [^64^Cu]Cl_2_ and [^64^Cu]-PSMA-617 in 62 prostate cancer patients. PET-CT postprostatectomy detection rate of a presumed local failure was compared with the current golden standard, multiparametric magnetic resonance (mpMR). It was found that both tracers demonstrated inferior performance to mpMR, and are not suitable as single imaging methods for assessing the prostatic bed in this context.^162^

### Brain Tumors

#### Anti-CD8 Minibody

CD8-positive (CD8+) T cells are a class of tumor-infiltrating lymphocytes (TILs) that have a crucial function in the immune response against tumors. Quantifying CD8+ T cells *in vivo* using radiolabeled fragments of anti-CD8 antibodies is a promising method to aid in the clinical advancement of new cancer immunotherapies and track the effectiveness of treatment.^163^

IAB22M2C is a humanized minibody, i.e., a genetically modified antibody fragment, that targets CD8. Due to their low molecular weight, good penetration, rapid clearance, and high accuracy, they are commonly used in tumor diagnosis and therapy. IAB22M2C was conjugated to a NOTA chelator and radiolabeled with ^64^Cu. One benefit of ^64^Cu, besides its long half-life, is that it does not build up in bone, which is a crucial consideration when performing immunological imaging on small animals.^163^

A 2021 report evaluated the imaging and monitoring of intratumoral CD8+ T lymphocytes in glioblastoma (GBM) tumors using [^64^Cu]-NOTA-anti-CD8 minibody PET. Human immune cells were taken from human peripheral blood mononuclear cells (PBMCs) to create a humanized immune system (HIS). The host mice employed in the study were severely immunodeficient mice created by the Central Institute for Experimental Animals, often known as NOG mice.^163^

To establish a quantitative correlation between the uptake of [^64^Cu]-NOTA-anti-CD8 and the presence of CD8+ T-cell infiltration, the uptake of [^64^Cu]-NOTA-anti-CD8 was evaluated using PET, while the absolute number of human CD8+ T cells in an organ rich in CD8+ T cells was measured using flow cytometry. Radiotracer retention correlated with the number of CD8+ T-cells in the spleen and tumor tissue, but negligible amounts were found in the normal brain. The work demonstrates the capability of [^64^Cu]-NOTA-anti-CD8 PET to measure the number of CD8+ T lymphocytes in both the peripheral and tumor-infiltrating regions of brain tumors.^163^

#### Anti-CD4 Minibody

Further research went on to study the application of [^64^Cu]-NOTA antibody conjugates in immunoPET to include other types of immune cells. This time, NOTA was combined with the humanized anti-CD4 minibody called IAB41. CD4-positive T cells are crucial in promoting the immunological response to tumors, along with CD8-positive T cells. There is increasing evidence to indicate that CD4-positive T cells also contribute to the immune evasion of brain tumors. The objective of the study was to assess the efficacy of a CD4-targeted minibody in detecting CD4+ T cells in peripheral organs and brain TILs using a PBMC HIS mouse model.^164^ It was found that [^64^Cu]-NOTA-IAB41 can be used to visualize CD4+ TILs in orthotopic PBMC HIS animal models of GBM. The radiotracer [^64^Cu]-NOTA-IAB41 is capable of identifying CD4+ T cells in both peripheral organs and brain tumors. The tested compound can identify human CD4+ T lymphocytes, regardless of their activation and functional states. Furthermore, no absorption of [^64^Cu]-NOTA-IAB41 was revealed in healthy brain tissue, indicating minimal background activity and that [^64^Cu]-NOTA-IAB41 is suitable for neuroimaging purposes. Further investigation is needed to determine the clinical capabilities of IAB41 immunoPET.^164^

#### Copper-64 Chloride

GBM tumors are derived from glial cells or their precursors and this group represents the most common primary brain cancer in adults. Due to tumor neovascularization, it is often accompanied by hypoxia, which causes an increase in the concentration of copper ions. In an *in vitro* study performed in GBM tumor spheroids, i.e., multicellular three-dimensional culture systems replicating the *in vivo* environment of tumor cells, [^64^Cu]Cl_2_ was found to significantly reduce spheroid growth and affect cell proliferation capacity. However, the uptake levels were not in agreement with the expression of CTR1 transporters in tumor cells, which suggests that another mechanism could be involved.^165^

A rare form of pediatric glioma is the pediatric-type diffuse high-grade glioma (PDHGG), which is the most common central nervous system neoplasms in children. PHDGGs have poor prognosis and since these tumors are virtually impossible to resect, their treatment relies on adjuvant radiotherapy combined with chemotherapy. [^64^Cu]Cl_2_ PETCT has been already identified as a promising procedure for identifying adult high-grade gliomas, often with high tracer uptake, as copper is an essential element in cellular metabolism and its turnover may be increased in tumor cells. A clinical pilot trial investigated the diagnostic and dosimetry features of [^64^Cu]Cl_2_ PET-CT in ten patients with PDHGG. It was found that [^64^Cu]Cl_2_ is well-tolerated and its dosimetric properties are reasonably favorable, with the liver being the limiting organ. It is selectively taken up by MRI contrast-enhancing/necrotic tumors with excellent target-to-background ratio. This finding suggests that BBB damage may be a prerequisite for the uptake of this tracer in the lesions. It was also found to accumulate in the tumors over time, suggesting that it may have therapeutic applications.^166^

### Neuroendocrine Tumors

One established therapeutic option for NETs that strongly express somatostatin receptors (SSTRs) is peptide receptor radionuclide therapy (PRRT). PRRT is a compelling treatment choice for administering cytotoxic radiation to cancer cells using a radiolabeled peptide that binds specifically to a cellular target.^167^

**Figure 8:**
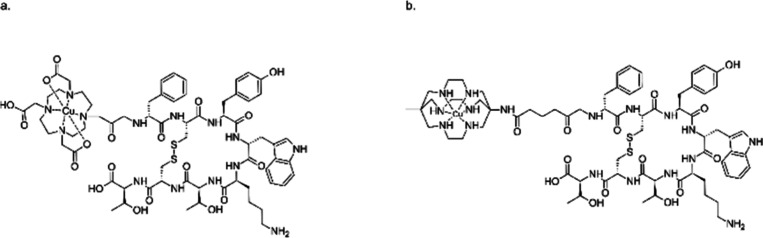
Structures of somatostatin analogues labeled with copper radioisotopes. (a) [Cu]-DOTATATE; (b) [Cu]-SARTATE.

Somatostatin is a cyclic peptide that plays a vital role in regulating exocrine, endocrine, and growth functions,^168^ and increased levels of SSTRs have been reported in individuals with NETs.^167,169^ One possible strategy for visualizing NETs uses ^64^Cu -radiolabeled DOTATATE as a PET radiotracer to highlight SSTRs; DOTATATE is a conjugate consisting of the macrocycle DOTA and Tyr3-octreotate (Figure 8a).^170−173^

A 2020 study described a phase III clinical trial including patients with SSTR-expressing NETs. The objective of the study was to determine the minimum dosage of [^64^Cu]-DOTATATE required to produce PET-CT images of sufficient quality for diagnosis, and to assess its diagnostic efficacy and safety. For diagnostic PET-CT pictures, the dose-ranging study found 148 MBq (4.0 mCi) was optimum. No negative incidents were recorded in relation to [^64^Cu]-DOTATATE, and no severe negative incidents were observed. The findings indicate that PET-CT imaging with 148 MBq of [^64^Cu]-DOTATATE is a secure and very precise method for diagnosing individuals with NET-positive conditions and tumors that express SSTR.^174^

Another study investigated the correlation between the density of tumor SSTR, observed using [^64^Cu]-DOTATATE, and both overall survival (OS) and progression-free survival (PFS). The study included a group of 128 patients with NETs of all World Health Organization categories, and tracked them for a period of over nine years. The primary result was that the SUV_max_ of [^64^Cu]-DOTATATE in tumor lesions was substantially linked to PFS, but not to OS. Nevertheless, the ability to predict PFS for each individual patient was only moderate. It appears that SSTR imaging has little to no significance in predicting individual patient prognosis comparted to [^18^F]-FDG PET-CT, which has been proven to substantially predict PFS and OS for different grades of NETs.^166^

In patients with NETs, SSTR-targeting PET imaging performed 1 h after injecting 200 MBq of [^64^Cu]-DOTATATE has been shown to be superior to other clinically accessible ^68^Ga PET and ^111^In SPECT SSTR-imaging modalities for lesion detection. A comparison was made between sets of [^64^Cu]-DOTATATE PET-CT images obtained one and 3 h after the injection of 200 MBq of [^64^Cu]-DOTATATE in 35 individuals with NETs. It was discovered that [^64^Cu]-DOTATATE PET-CT performs exceptionally well under these conditions, with no discernible variation in the quantity of lesions found.^175^

The synthesis and preclinical evaluation of [^64^Cu]-SARTATE, i.e., [^64^Cu]-MeCOSar conjugated to Tyr3-octreotate, (Figure 8b) using a cage amine ligand known as MeCOSar was described in 2014.^176^ MeCOSar offers the advantage that copper(II) SAR complexes demonstrate greater stability than copper(II) DOTA complexes.^177^

In 2019, the safety and effectiveness of [^64^Cu]-SARTATE to detect diseases at both early and late stages of imaging was evaluated in humans. Significant accumulation and retention of lesions were found at all imaging time-points, *viz*. Thirty minutes, 1 h, 4 h, and 24 h following the injection of [^64^Cu]-SARTATE. A comparative analysis was conducted between the [^64^Cu]-SARTATE PET scans and the [^68^Ga]-DOTATATE images, with all patients being selected based on a positive [^68^Ga]-DOTATATE scan. For neuroendocrine neoplasia, PET-CT using ^68^Ga-labeled somatostatin analogues is quickly replacing other methods as the gold standard. However, due to the rapid physical decay of the radionuclide, treatment is associated with logistical and regulatory issues.^177^

All 10 patients demonstrated either comparable or better lesion identification with [^64^Cu]-SARTATE PET-CT acquired after 4 h compared to [^68^Ga]-DOTATATE PET-CT obtained after 1 h. Also, [^64^Cu]-SARTATE treatment did not elicit significant side effects. In conclusion, [^64^Cu]-SARTATE is a safe option for diagnostic tests and prospective dosimetry for [^67^Cu]-SARTATE due to its high late-retention in tumors and efficient clearance from the liver.^177^

A 2020 study examined the effectiveness of [^67^Cu]-SARTATE as a treatment for NETs in a preclinical model. The study attempted to compare the efficacy of [^67^Cu]-SARTATE with the conventional PRRT agent, [^177^Lu]-DOTATATE in a mouse model with AR42J (rat pancreatic exocrine) tumors. A single injection of [^67^Cu]-SARTATE (5 MBq) reduced cancer development by 75% after 7 days compared to the control group, whereas ^177^Lu-DOTATATE reduced tumor growth by 89%. Both [^67^Cu]-SARTATE and [^177^Lu]-DOTATATE showed equal extension of survival. The administration of a total of 30 MBq of either [^67^Cu]-SARTATE or [^177^Lu]-DOTATATE, in two separate 15-MBq doses given 2 weeks apart, significantly increased survival compared to a single dose of 30 MBq. The study shows that the [^67^Cu]-SARTATE possesses anticancer activity in the AR42J tumor model, indicating that this new drug should be explored in clinical trials for the treatment of NETs that express somatostatin.^170^ Currently, the safety and effectiveness of [^67^Cu]-SARTATE are under evaluation in a clinical study in children diagnosed with high-risk neuroblastoma (clinical trial ID NCT04023331).

### Human Immunodeficiency Virus

Research aimed at identifying a cure for Human Immunodeficiency Virus (HIV) has focused on identifying the specific locations and mechanisms of HIV persistence in individuals undergoing antiretroviral therapy.^178^ A new method using immunoPET was developed in 2015 to detect total-body simian immunodeficiency virus (SIV) replication in viremic monkeys, using a ^64^Cu-labeled SIV Gp120 antibody.^179^ A small percentage of HIV-1 infected individuals exhibit significant antiviral activity, primarily due to broadly neutralizing antibodies (bNAbs), such as 3BNC117. 3BNC117 can neutralize a wide range of HIV-1 envelope variants by targeting the CD4 binding region.^180^ The chemical underwent phase I clinical trial, during which it was demonstrated to be both safe and effective in lowering HIV-1 viremia.^181^

A phase I clinical trial was conducted to explore the use of ^64^Cu PET in detecting HIV reservoirs in humans. During the study, the NHS ester of MeCOSar was reacted with 3BNC117, and the resulting product, MeCOSar-3BNC117, was isolated through purification. This compound was then labeled with ^64^Cu to produce [^64^Cu]-MeCOSar-3BNC117. The compound was administered to both participants who were receiving intermittent antiretroviral treatment, as well as HIV-uninfected patients. The presence of HIV Envelope-containing tissues was identified using PET and MRI imaging techniques at one, 24 and 48 h after administration. The administered [^64^Cu]-MeCOSar-3BNC117 exhibited comparable pharmacokinetics and biodistribution between controls, viremic, and aviremic patients. No discernible difference in HIV binding or neutralization was observed *in vitro* among the three compounds, namely 3BNC117, MeCOSar-3BNC117, and [^64^Cu]-MeCOSar-3BNC117, throughout the course of the study. The findings suggest that the [^64^Cu]-MeCOSar-3BNC117 compound is safe, since no significant side effects were identified; however, no HIV envelope expression was detected due to the inability of [^64^Cu]-MeCOSar-3BNC117 to differentiate between uninfected and infected clinical trial participants. Throughout the study, it was observed that the MeCOSar chelator and ^64^Cu had no significant impact on the functionality of 3BNC117. Further research should explore different radiolabels with a longer half-life, such as zirconium.^178^

### Melanoma

Human melanotic and amelanotic melanoma are characterized by elevated levels of the G protein-coupled receptor melanocortin-1 receptor (MC1R). Peptides of alpha-melanocyte-stimulating hormone (α-MSH) have the ability to bind to MC1Rs with affinities in the nanomolar range.^182^

**Figure 9:**
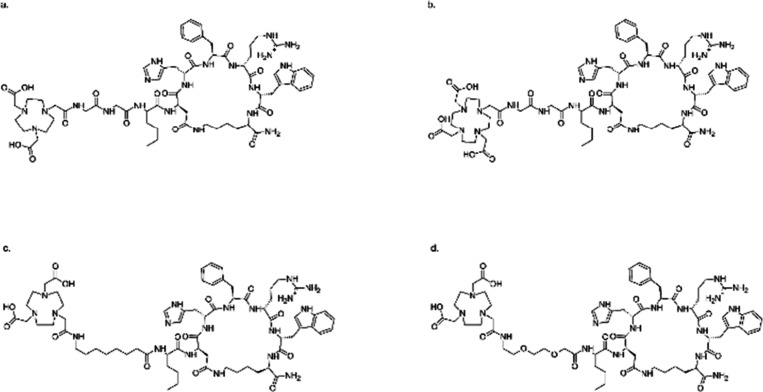
Structures of tracers incorporating a MSH targeting moiety: (a) NOTA-GGNle-CycMSH_hex_, (b) DOTA-GGNle-CycMSH_hex_, (c) NOTA-AocNle-CycMSH_hex_, (d) NOTA-PEG_2_Nle-CycMSH_hex_.

A 2012 study examined the melanoma targeting and imaging properties of [^64^Cu]-NOTA-GGNle-CycMSHhex (Figure 9a) and [^64^Cu]-DOTA-GGNle-CycMSHhex (Figure 9b) in B16/F1 melanoma-bearing C57 mice. These are second-generation versions of the lactam-cyclized α-MSH peptides - CycMSHhex {c[Asp-His-DPhe-Arg-Trp-Lys]-CONH_2_}. It was found that [^64^Cu]-NOTA-GGNle-CycMSHhex exhibited significantly greater melanoma uptake and decreased renal and liver uptake in comparison to [^64^Cu]-DOTA-GGNle-CycMSHhex. Consequently, [^64^Cu]-NOTA-GGNle-CycMSHhex was proposed as a potential radiolabeled peptide for use in PET melanoma imaging and treatment.^183^

The tumor targeting and biodistribution of NOTA-conjugated lactam-cyclized α-MSH peptides in B16/F10 melanoma-bearing C57 mice were subsequently investigated.^182,184^

The initial investigation assessed the effects of replacing the Gly-Gly (GG) linker with either an 8-aminooctaoic acid (Aoc) or polyethylene glycol (PEG) linker on tumor targeting and biodistribution. NOTA-PEG2Nle-CycMSHhex (Figure 9d) and NOTA-AocNle-CycMSHhex (Figure 9c) were radiolabeled with ^64^Cu. It was found that replacing the GG linker with an Aoc or PEG linker can have a significant impact on uptake by melanoma, with [^64^Cu]-NOTA-GGNle-CycMSHhex and [^64^Cu]-NOTA-PEG2Nle-CycMSHhex demonstrating greater melanoma uptake compared to [^64^Cu]-NOTA-AocNle-CycMSHhex. Two hours after injection, [^64^Cu]-NOTA-PEG2Nle-CycMSHhex demonstrated high tumor to normal organ uptake ratios, and the B16/F10 melanoma lesions were distinctly visible via PET imaging^184^

Further investigation utilized ^67^Cu to radiolabel NOTA-PEG2Nle-CycMSHhex and NOTA-GGNle-CycMSHhex. [^67^Cu]-NOTA-PEG2Nle-CycMSHhex demonstrated superior tumor uptake compared to [^67^Cu]-NOTA-GGNle-CycMSHhex across all imaging time probes, with particularly high uptake ratios from tumor to normal organs observed 2 h after injection. At 4 h after administration, normal organ uptake was below 2.6% ID/g, with the exception of kidney uptake. At 4 h after injection, B16/F10 melanoma lesions were clearly visible via SPECT imaging employing [^67^Cu]-NOTA-PEG2Nle-CycMSHhex as the imaging probe.^182^

The research indicates that [^67^Cu]-NOTA-PEG2Nle-CycMSHhex SPECT and [^64^Cu]-NOTA-PEG2Nle-CycMSHhex PET exhibited favorable tumor targeting and biodistribution characteristics, which highlight their potential as MC1R-targeted therapeutic peptides for the treatment of melanoma.^182,184^

## Summary

The use of radioactive copper isotopes is a promising step in the development of nuclear medicine. The possibility of using a single element for a range of nuclear medicine applications is an almost ideal solution for future nuclear medicine specialists, radiopharmacists and scientists developing new targeted ligands. This goal is becoming steadily closer thanks to the joint work of nuclear physicists, chemists, pharmacists and physicians. These efforts have already borne fruit thanks to the dynamic development of copper isotope production via nuclear reactions, photonuclear synthesis, and the development of new isotope generators. In addition, the research has yielded new modifications of chelating compounds capable of rapid complexation of copper ions, allowing them to form stable *in vivo* complexes conjugated with new targeting ligands. Finally, numerous studies have demonstrated that these substances are effective in potential medical applications and offer further promise for use in many different areas of medicine.

## Abbreviations

α-MSH: α-melanocyte-stimulating hormone
Aβ: amyloid-β protein
AD: Alzheimer’s disease
Aoc: 8-aminooctaoic acid
BBB: blood–brain barier
BBN: bombesin
BFCA: bifunctional chelating agent
bNAbs: broadly neutralizing antibodies
BTSC: bis(thiosemicarbazone)
DOE-IP: Department of Energy Isotope Program
DOTA: 2,2′,2″,2‴-(1,4,7,10-tetraazacyclododecane-1,4,7,10-tetrayl)tetraacetic acid
EDTA: ethylenediaminetetraacetic acid
FDG: 18F-fluorodeoxyglucose
GBM: glioblastoma
GG: Gly-Gly linker
GRPR: gastrin-releasing peptide receptor
HIS: humanized immune system
HIV: human immunodeficiency virus
IAEA: International Atomic Energy Agency
*K*_ML_: constant of complex formation
*k*_reverse_: constant of complex dissociation
LinAcs: linear electron accelerators
MC1R: melanocortin-1 receptor
MCC: maleimidomethyl cyclohexane-1-carboxylate
mpMR: multiparametric magnetic resonance
MRI: magnetic resonance imaging
NETs: neuroendocrine tumors
NHS: *N*-hydroxysuccinimydyl
NIRS: National Institute of Radiological Sciences
NOTA: 2,2′,2″-(1,4,7-triazacyclononane-1,4,7-triyl)triacetic acid
OS: overall survival
PBMCs: peripheral blood mononuclear cells
PDHGG: pediatric-type diffuse high-grade glioma
PEG: polyethylene glycol
PET: positron emission tomography
PET-CT: positron emission tomography combined with computed tomography
PFS: progression-free survival
PRRT: peptide receptor radionuclide therapy
PSMA: prostate specific membrane antygen
ROS: reactive oxygen species
SAR: sarcophagine
SIV: simian immunodeficiency virus
SPECT: single-photon emission computed tomography
SSTRs: somatostatin receptors
SUV_max_: standardized uptake value
T-DM1: trastuzumab-emtansine
TACN: 1,4,7-triazacyclononane
TETA: 1,4,8,11-tetraazacyclotetradecane-1,4,8,11-tetraacetic acid
TILs: tumor-infiltrating lymphocytes
WD: Wilson’s disease
